# Training People to Think in Opposites Facilitates the Falsification Process in Wason’s Rule Discovery Task

**DOI:** 10.3390/jintelligence11050091

**Published:** 2023-05-11

**Authors:** Erika Branchini, Roberto Burro, Ivana Bianchi

**Affiliations:** 1 Department of Human Sciences, University of Verona, Lungadige Porta Vittoria 27, 37129 Verona, Italy; 2 Section Philosophy and Human Sciences, Department of Humanities, University of Macerata, Via Garibaldi, 20, 62100 Macerata, Italy

**Keywords:** Wason’s rule discovery task, 2-4-6 task, opposites, contrast, facilitation training

## Abstract

With reference to Wason’s 2-4-6 rule discovery task, this study investigated the effects of a simple training session that prompted participants to “think in opposites”. The results showed a significant improvement in performance under the training condition when compared to the control condition, both in terms of the proportion of participants who discovered the correct rule and how quickly it was discovered. An analysis of whether or not participant submitted test triples formed of descending numbers showed that fewer participants under the control condition considered ascending/descending to represent a critical dimension and, in any case, this occurred later (that is, after more test triples) than in the training condition. These results are discussed in relation to previous literature showing improvements in performance that were prompted by strategies involving “contrast” as a critical factor. The limitations of the study are discussed, as well as the benefits of a training program like this, which is non-content related.

## 1. Introduction

Wason’s rule discovery task (Wason 1960) is one of the classic problems in the psychology of reasoning. Starting from a seed triple (2-4-6) that conforms to a rule that the experimenter has in mind, participants are asked to discover the rule. They can make hypotheses and test them by generating triples (usually referred to as test triples). The experimenter provides feedback for each test triple in terms of whether or not it conforms to the rule. When participants believe that they have guessed the rule, they announce what they think it is. What is interesting in this task is that it gives the experimenters an opportunity to observe the line of thought that participants follow when testing their hypotheses.

Although formulated more than 60 years ago, this problem still stimulates empirical research (Cooper et al. 2022; Hegarty 2017; Mayo et al. 2014; Ragni et al. 2018; Rusconi et al. 2012; Sauerland et al. 2020). We were particularly interested in the type of prompts that might improve the efficacy of the hypothesis testing procedure participants tend to use by default (see paragraph below). Specifically, the aim of the study presented in this paper was to explore the effects of a training program that explicitly stimulated the participants to “think in opposites”. This idea is partially in line with two previous pieces of research (Gale and Ball 2012; Rossi et al. 2001), but the kind of facilitation used in the present study differs from these two previous studies (see method Section 2.1). Furthermore (as discussed in Section 1.2 and Section 3), the theoretical premise that the facilitation in this study is based on, in part connects to, but in part differs from, the theoretical ideas presupposed by the works cited above. In this sense, we trust that this study reveals the connections between the research into opposites (Bianchi et al. 2011a, 2011b, 2013, 2017a, 2017b, 2017c) and that into contrast sets and the iterative counterfactual strategy (Oaksford and Stenning 1992; Oaksford and Chater 1994a, 1994b; Oaksford 2002).

### 1.1. Some Ways to Facilitate the 2-4-6 Task

Despite the apparent simplicity of Wason’s 2-4-6 task, success rates for first attempts are quite low, around 20% (e.g., Tukey 1986; Wason 1960; Wharton et al. 1993). The rule the experimenter has in mind is simply “ascending numbers”, whereas participants typically think of more restrictive rules, such as “numbers increasing by intervals of two”.

As far as the testing strategy is concerned, participants tend to test triples that are compatible with their initial hypothesis instead of testing counterexamples or triples that are not compatible with their hypothesis (e.g., Gale and Ball 2012; Evans 2016; Ragni et al. 2018; Sauerland et al. 2020; Wason 1960). We might compare this to Popper’s famous example in which the participants keep looking for white swans in order to prove the hypothesis that all swans are white rather than trying to find a black swan that would contradict the rule. Thus, in the present case, if the participant’s initial hypothesis is “even numbers increasing by intervals of two”, in testing, they will persevere in testing triples that conform (such as 6-8-10, 10-12-14, or 40-42-44, etc.). These triples are, in fact, a subset of the correct rule (i.e., ascending numbers) and therefore they receive positive feedback. Positive feedback strengthens the participants’ beliefs in their hypothesis, which, although partially correct, is not the right answer—this type of strategy results in low success rates.

Various studies have tried to encourage the use of falsification strategies by changing the instructions for the task. A number of these studies are of particular interest for the present paper since they call into play a kind of reasoning that somehow relates to the notion of thinking in opposites (see also Section 1.2).

Gorman and Gorman (1984; see also Gorman et al. 1984) started from Tweney et al.’s (1980) version of Wason’s task and added explicit instruction to follow a disconfirmation strategy. “Suppose that 5-7-9 is consistent with the rule. You would then have evidence that your hypothesis (e.g., three equal numbers) is wrong. Notice that this strategy allows you to get evidence about whether your hypothesis is correct by testing number triples that you don’t think will fit the rule” (Tweney et al. 1980, p. 114). Under this condition, the participants performed better than when they were not given any explicit suggestions. They also performed better compared to when a confirmatory strategy was used. “Suppose that 8-8-8 is consistent with the rule. You would then have evidence supporting your hypothesis. Notice that this strategy allows you to get evidence that your hypothesis is correct by thinking up number triples that you think will fit the rule” (Tweney et al. 1980, p. 114).

Improvements in performance were also noted in another study in which the participants were presented with a slightly modified version of Wason’s original task. The variation consisted of presenting the 2-4-6 seed triple as a counterexample of the rule (i.e., a sequence that did not follow the rule) rather than giving them an example that conformed to the rule (Rossi et al. 2001).

Finally, a third group of studies that are relevant here concerns various versions of the dual goal instruction task (Tweney et al. 1980). This is a variant of Wason’s original task, and it is traditionally associated with better success rates. In this task, the participants are required to discover not one but two rules: DAX (i.e., the same as in Wason’s original task, that is, any sequence of numbers in ascending order) and MED (all other triples). The rationale underlying this new version was based on the consideration that in Wason’s classic version of the task, the test triples proposed by the participants that did not conform to the rule were classified as being wrong, perhaps preventing them from using this negative feedback in a productive way. To avoid this, Tweney and colleagues changed the instructions so that all of the test triples would be classified as instances of one rule (DAX) or the other (MED) rather than simply stating that they were right or wrong. In this new version, 60% of the participants guessed the DAX rule on their first attempt (Gale and Ball 2006; Gorman et al. 1987; Tukey 1986; Vallée-Tourangeau et al. 1995; Wharton et al. 1993).

Gale and Ball (2006, 2009, 2012) explored the mechanism underlying this improvement in performance in a series of studies in which the experimenters used different seed triples as examples of the MED rule. In the last study in particular (Gale and Ball 2012), the authors selected the seed triples to be tested based on the notion of the contrast class (Oaksford and Stenning 1992). According to Oaksford (2002, p. 140), if we are told, for example, that John *is not* drinking coffee, we are led to imagine that he is drinking tea, hot chocolate, or Ovaltine; we do not assume that he is drinking whisky, water, or cola. In other words, we do not consider all of the possible elements that could form the logical complement set relating to coffee (i.e., all drinks that are not coffee) but only take into account some of the psychologically relevant members of that set (i.e., other hot drinks). Gale and Ball (2012) use the idea of the contrast class to explore the effect of giving participants different types of seed triples as examples of the MED rule. They found that the MED triple that worked best as a facilitating clue was 6-4-2 (as compared to two other triples, 4-4-4 and 9-8-1). This finding confirmed their hypothesis that “the seemingly oppositional nature of DAX and MED provides participants with cues (via triple generation) that soon facilitate insight into the ascending nature of the DAX rule. In particular, receiving MED feedback for a descending triple such as 6-4-2 in the dual goal task is argued to alert reasoners to the realization that MED and DAX triples may fall at opposite poles of the dimension descending versus ascending” (cit, p. 410). In other words, the triple 6-4-2 makes the relationship between the two rules psychologically evident. In fact, 6-4-2 contrasts with the 2-4-6 seed triple (an example of the DAX rule) with respect to only *one* dimension, and it is this dimension that is, in fact, relevant (i.e., increasing vs. decreasing numbers). The other triples (4-4-4 and 9-8-1) contrast with the seed triple along dimensions that are not relevant; the sequence 4-4-4 encourages participants to focus on the contrast between triples consisting of equal versus unequal numbers. Meanwhile, the 9-8-1 triple orients their attention towards the contrast between irregular versus regular intervals between numbers in addition to the contrast between ascending versus descending numbers, which is, in fact, the correct target characteristic.

### 1.2. Rationale for Testing the Effects of Training Reasoners to “Think in Opposites”

All of the facilitations described in the previous section encouraged the participants to consider not only the triples that were compatible with their initial hypothesis but also any alternatives to it (Evans 2016; Ragni et al. 2018). This means that the triples either contrasted with the initial hypothesis (as in the case of the 6-4-2 example of the MED rule used by Gale and Ball 2012) or were counterexamples of it (Rossi et al. 2001). The study presented in this paper tested the effects of yet another way of prompting participants to use contrast as a key strategy in the hypothesis-testing phase. In particular, our aim was to understand whether participants taking part in a standard version of Wason’s rule discovery task would benefit from training that explicitly advised them to think in opposites when they generated test triples to verify their hypotheses.

There were three main reasons why we considered this type of training to be worth testing. First, it is simple for the participants to follow a prompt to think in opposites. Opposition is implied in various reasoning processes (more than we believe—for a review, see Branchini et al. 2021) and is spontaneously used in everyday reasoning to imagine alternatives to reality by both children (e.g., Beck et al. 2006; Fitzgibbon et al. 2019; Rafetseder et al. 2010) and adults (Byrne and McEleney 2000; Byrne 2016, 2018). The fact that we find it easy to use opposites when thinking about alternatives is not surprising if we consider that opposition constitutes a basic relationship that structures human conceptual spaces (e.g., Gärdenfors 2000, 2014). People have an intuitive idea of the concept despite being unable to formulate a clear definition of the requisites for two meanings or configurations to be opposite. This has emerged from language studies (e.g., Jones 2002, 2007; Paradis et al. 2009) and perceptual studies that have shown that the perception of opposites is essential to spatial perception (e.g., Bianchi et al. 2011a, 2013, 2017a; Bianchi and Savardi 2018). There is also evidence that humans possess an intuitive idea of two opposite visual or acoustic configurations (Bianchi et al. 2017b, 2017c), as well as of two opposite postures or gestures (e.g., Bianchi and Savardi 2008a, 2008b; Bianchi et al. 2014). The primary intuition of opposition has its roots in infants’ pre-verbal categorization (e.g., Casasola 2008; Casasola et al. 2003). Because humans possess an intuitive idea of this concept, we expected that it would be simple for the participants to follow a prompt to think in opposites when they were testing alternative hypotheses in Wason’s triple task. During training, we explained what we meant by making reference to the visual characteristics of simple bi-dimensional shapes (see method, Section 2.1). The aim was for them to apply the same strategy when dealing with numbers (for which opposition was characterized by dimensions such as even-odd, small-large, ascending-descending, and same-different increments, etc.).

The second reason for testing this strategy is linked to the fact that two of the successful facilitations described in the previous paragraph (namely, Gale and Ball 2012 and Rossi et al. 2001) presupposed a notion of contrast and/or opposition. In particular, in Gale and Ball’s study, the relationship between the two triple seeds given to participants and associated with the improvement in success rates (i.e., 2-4-6 for DAX and 6-4-2 for MED) is evident. The oppositional relationship between the two series is salient and clear, even though this is not explicitly referred to by the experimenters in terms of “opposites” in their instructions.

In our study, the idea of opposition was made explicit, and the suggestion to use this idea was not hinted at implicitly but was part of an explicit training session. The participants were advised to concentrate on all of the properties of the seed triple and then identify the opposite of these properties in order to use them to test their hypotheses. The decision to use explicit training rather than a hint was based on the results of a number of previous studies that had investigated the effects of prompting participants to think in opposites in visuospatial problem-solving (Bianchi et al. 2020; Branchini et al. 2015a, 2015b, 2016, 2021). The results of these studies, in fact, gave us a third reason to modulate the experimental task in the present study (i.e., explicit training to think in opposites). The participants’ performance in visuospatial problems improved when they were explicitly trained to start their search for a solution by carefully focusing on all of the properties that characterized the elements of the problem (the first step) and then identifying the opposite of these properties (the second step). Following this, in the third step, they were given three examples that showed how thinking in opposites could help them to break the constraints caused by their initial mental representation of the problem and, thus, find the correct solution (Bianchi et al. 2020; Branchini et al. 2016). In the case of visuospatial problems, the explicit training session was more effective than an implicit hint to use contraries. This latter strategy (see Branchini et al. 2015a, 2015b) basically consisted of the first two steps described above (i.e., an analytical listing of the properties of the visuospatial problems and the identification of their opposites), but there was no third step in which the application of thinking in opposites was explicitly displayed and exemplified. A certain awareness of why listing opposites could be relevant was associated with better results. Based on these previous findings, the present study aimed to test the effect of using the same strategy in Wason’s rule discovery task, and we chose to use an explicit training program, modeling it on that used with the visuospatial problems (Bianchi et al. 2020; Branchini et al. 2016).

An interesting characteristic of this training, and a further difference from the two previously mentioned studies (i.e., Gale and Ball 2012; Rossi et al. 2001), is that the facilitation given was not content related. That is, the facilitation did not make reference to a specific triple but rather suggested a general procedure to follow. Training is often procedural, as it advises a reasoning strategy, while hints are usually content related. As the training used in this study was not content related, it is generalizable to other contexts. We will return to this in the final discussion.

## 2. The Study

The aim of this study was to investigate whether participants taking part in a standard version of Wason’s rule discovery task would benefit from training that advised them to think in opposites when they generated test triples to verify their hypotheses. The training condition was compared to a control condition in which no facilitation was provided.

There were four hypotheses to test:

*(1) Success rates*. We hypothesized that training would increase the number of participants who discovered the correct rule.

*(2) Effectiveness of the attempts made*. We wanted to ascertain whether training would result in the correct response emerging earlier. It was possible that the participants in the two conditions would come up with the same overall number of correct responses but that the correct response would be announced earlier under the training condition. “Earlier”, in this case, was defined both in terms of the time it took to discover the rule and with reference to the number of attempts made. In other words, did participants discover the solution on the first, second, or third attempt? It could be that under the training condition, the participants might take more time before announcing the correct rule but be more efficient in that they would more frequently find the correct rule on the first attempt.

*(3) Identification of the critical dimension*. We wondered whether participants exposed to the training would be able to identify the critical characteristic (i.e., an ascending series of numbers) more often compared to the control condition. We also wondered whether they would identify it earlier and whether once identified, they would actually use it more often to solve the task.

*(4) Flexibility*. The study aimed to ascertain whether there would be any differences between the training and control conditions in terms of whether the participants reiterated test triples that focused on the same property versus test triples focusing on different properties. The latter would indicate that the participants were exploring other alternatives in the hypothesis-testing phase.

The answers to questions three and four make it possible to put forward some specific hypotheses regarding the level on which the proposed strategy impacts the default hypothesis testing procedure (as reflected in the control condition). This may well inspire future studies, as discussed in the final section.

### 2.1. Method

#### 2.1.1. Participants

A total of 120 participants (57 males and 63 females; *M*_age_ = 30.16, *SD* = 12.51) took part in the study. Participation was voluntary and subject to informed consent. The study conformed to the ethical principles of the Declaration of Helsinki (World Medical Association 2013) and was approved by the Ethics Committee of the University of Macerata (prot. n. 37413/2023).

#### 2.1.2. Procedure

Each participant took part in the study individually. They were randomly assigned to one of the two conditions (i.e., training or control). Under the control condition, the participants were not given training and were simply presented with Wason’s rule discovery task. We used the standard version of Wason’s task in which participants have to discover a single rule starting from the seed triple 2-4-6.

Using the booklet provided, the participants were asked to write their test triples in the exact order in which they wished to submit them. They received feedback for each test triple that informed them if it conformed to the correct rule. When the participants thought they had identified the correct rule, they were asked to write this down and also tell the experimenter. If the rule was correct, the experiment finished; if not, they were given two more attempts (see Evans 2016).

Under the training condition, a strategy based on opposition was explained to the participants, and they were encouraged to apply this strategy when they generated test triples. The experimenter read the explanation out loud and also gave them a printed sheet. The strategy was explained with reference to a series of shapes rather than numbers in order to avoid mentioning properties such as odd, even, ascending, descending, constant intervals, and so on that would later be crucial to Wason’s task. Here are the instructions given to the participants, which were read aloud by the experimenter:

“This strategy can be used to generate the test triples that will help you to discover the rule for Wason’s problem. To explain the strategy, I will refer to triples made up of shapes, but in the task, numbers are used.

Below is a triple set of shapes that conforms to the rule I have in mind. Look carefully at the three shapes and think about the properties they have in common.

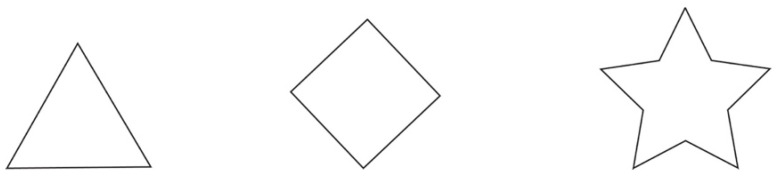

For instance:

- all of the sides are straight;
- they are all plane figures (2D);
- they are all symmetrical figures;
- hey are all closed figures;
- the figures are aligned with each other;
- they are all empty figures;
- they have all different shapes (i.e., they are different from each other).

Since you have to discover the rule I have in mind, you need to figure out which of these characteristics is relevant to the rule. How can this be done? We suggest “thinking in opposites”, which means focusing on one characteristic at a time and identifying its opposite. For instance, if all of the shapes have *straight* sides, you might ask yourself whether this characteristic is crucial. To verify this, carry out a test with a triple set composed of shapes that have *curved* sides. If the feedback indicates that the triple you have proposed is still a good example of the rule, this means that it is irrelevant whether the shapes have straight or curved sides.

You should then focus on another characteristic and test it in a similar way (e.g., they are all *bi-dimensional* shapes). To verify whether this feature is crucial, generate a triple with *three-dimensional* shapes, and if this is still a good example of the rule I have in mind, it means that this, too, is an irrelevant characteristic.

Another characteristic to test might concern the fact that all of the shapes are *symmetrical*. If you generate a triple set with *asymmetrical* shapes, and it is still a good example of the rule I have in mind, it again means that this is an irrelevant characteristic.

I hope you have understood what to do. If so, I will now show you the triple set of numbers that represents the rule you are asked to find. Focus on one characteristic of the set at a time, write it down, and then think of its opposite; that is, find out whether a triple set that is opposite in terms of the characteristic you are focusing on still conforms to the rule or not. Write this test triple (and also the feedback you receive) on your response sheet. You can test as many triples as you would like, noting them in the order that you test them. Once you think you have identified the rule, say it aloud (and also write it), and I will tell you if it is correct. If it is not, you can continue to test using other triples, but remember that you only have three chances to guess the rule. Are you ready? The test triple is 2-4-6”.

Response times were measured in both the training and control conditions, starting after the experimenter had finished reading the instructions. In both conditions, the experiment lasted less than 15 min.

#### 2.1.3. Materials

The materials consisted of a booklet made up of two sheets. The first page was for the participant’s personal information (gender and age) and the instructions for the task. On the second page, the seed triple (2-4-6) was printed in bold at the top, and the remaining space was divided into three sections that corresponded to the three attempts that the participants were allowed to make to guess the rule. To the left of each of these sections, there was space to write the test triples (in order) and the feedback they received. The space on the right was for the participants to write what they thought the rule was. There were headings at the top of each section so that it was clear where participants were supposed to write their test triples and feedback and where to write what they thought the rule was.

#### 2.1.4. Data Analyses

Linear models (stats R-package, version 4.2.3, R Core Team 2023) and generalized linear models (lme4 R-package, version 1.1.31, Bates et al. 2015) were used to analyze the effects of the two conditions (training and control) on the various dependent variables considered. The power of each analysis was tested using the WebPower R-package (version 0.9.2, Zhang and Mai 2023). Mosaic plots (vcd R-package, version 1.4.10, Meyer et al. 2006) were used to represent the counts relating to the number of attempts to guess the rule made by the participants under the two different conditions. A mosaic plot is a graphical, proportional representation of a contingency table. It consists of tiles corresponding to the cells of the contingency table which are determined by recursively dividing a square horizontally and vertically. A blue tile indicates that there are more observations in that tile than would be expected under the null model (independence). A red tile indicates there are fewer observations than expected. Pearson residuals (conventionally represented to the right of the mosaic) are defined as the standardized distances between the observed and expected responses.

## 3. Results

The results are described in relation to each of the hypotheses discussed in the study, respectively.

**Figure 1:**
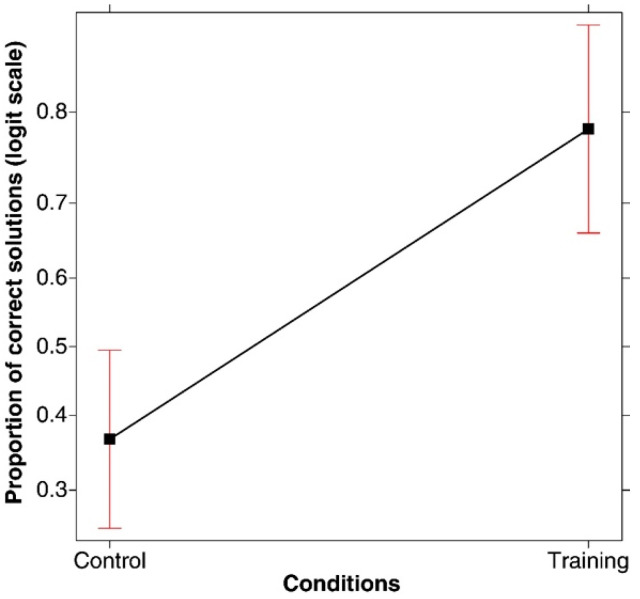
Main effect of correct solutions in the two conditions. Error bars represent the 95% confidence interval.

**(1) Success rate.** A generalized linear model (logit link function, binomial family) applied to the proportion of correct solutions under the two conditions revealed that training was associated with better performances (*χ*^2^(1, *N* = 120) = 22.067, *p* < .001; *R-squared* = 0.178; *power* (*alpha* = 0.05) = 0.998). As shown in Figure 1, the proportion of correct solutions was significantly higher under the training condition compared to the control condition. In fact, 78.33% of participants found the correct solution in the training condition compared to only 36.66% in the control condition.

**Figure 2:**
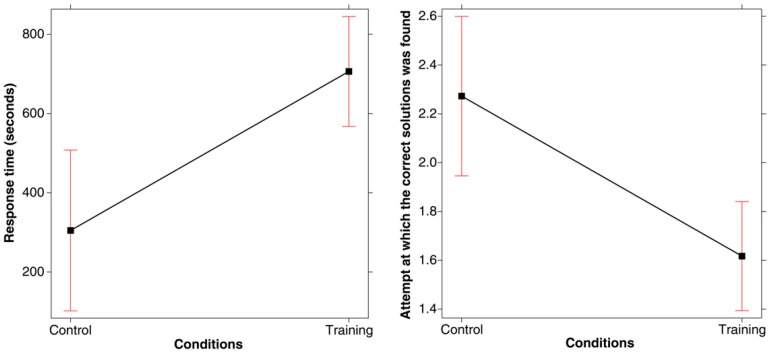
(**Left Panel**) Main effect of Response time associated with determining the correct solution in the two conditions. (**Right Panel**) Main effect of Attempt (i.e., first, second, or third) at which the correct solution was found in the two conditions (average value; the participants had a maximum of three attempts at their disposal). In both graphs, error bars represent the 95% confidence interval.

**(2) Efficacy of the search procedure: the point at which the correct solution emerged**. We conducted a linear model of the response times associated with finding the correct solution. The analysis was only performed on the subset of responses consisting of a correct solution with Condition as the fixed effect. The analysis revealed that training had a temporal cost: finding the correct solution in the training condition took longer than in the control condition (*F*(1, 67) = 10.630, *p* = .002; *R-squared* = 0.137; *power* (*alpha* = 0.05) = 0.991)—see Figure 2 (left panel).

**Figure 3:**
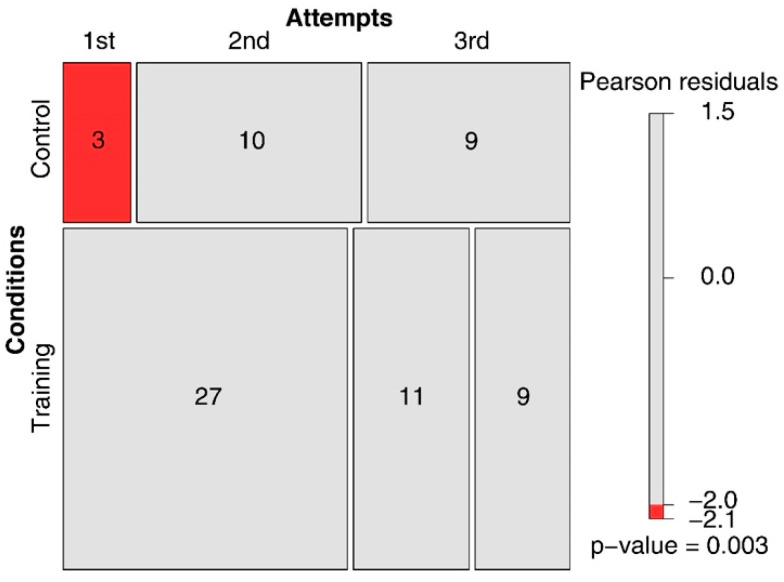
Mosaic plot of the number of correct solutions on the three attempts for the two conditions studied (Training and Control).

However, with regard to the attempt at which the correct solution was found (of the three attempts permitted), the participants in the training condition were successful earlier than those in the control condition. This was discovered after recoding the correct responses in terms of the attempt at which the correct rule was discovered (i.e., 1st, 2nd, or 3rd) and then conducting a linear model with these data. A main effect of Condition emerged (*F*(1, 67) = 10.937, *p* = .001; *R-squared* = 0.140; *power* (*alpha* = 0.05) = 0.992), which is represented in Figure 2 (right panel). Training improved success rates relating to the first attempt. Indeed, out of the 47 correct solutions found in total under the training condition, 57.4% were found on the first attempt, 23.40% on the second attempt, and only 19.14% on the third attempt. Conversely, in the control condition, out of the 22 correct solutions found in total, only 13.63% were found on the first attempt, 45.45% on the second attempt, and 40.90% on the third attempt. As shown in the mosaic plot in Figure 3, the significant difference concerns, in particular, the correct solutions found on the first attempt.

**(3) Identifying the critical property (ascending-descending)**. Under the training condition, the participants were required to identify a property to focus on as part of the procedure and then produce a triple by thinking in terms of the opposite of this property. Every time participants produced a test triple, they verbally stated the property they were testing. Under the control condition, two independent judges inferred the property being tested by identifying the property that varied in the test triple in relation to the seed triple. For instance, the triple 3-5-7 was coded to test the hypothesis that odd versus even numbers represented the critical property. The inter-rater reliability agreement between the two independent judges, measured by Cohen’s kappa coefficient, was good (i.e., equal to 0.82).

**Figure 4:**
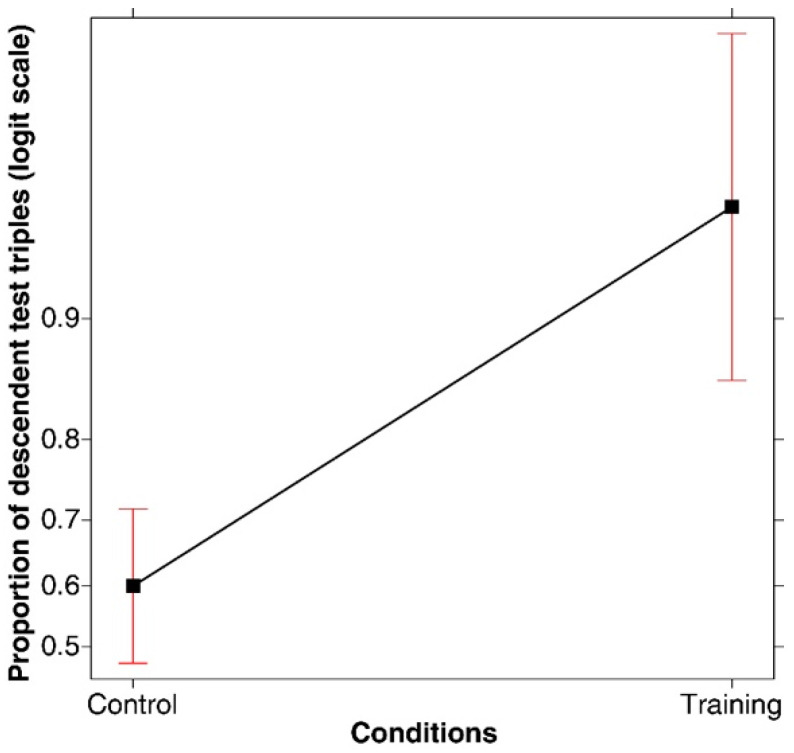
Main effect of the proportion of participants submitting a descending test triple in the control and training conditions. Error bars represent the 95% confidence interval.

Independently of whether the participants discovered the rule or not, we studied, first of all, whether the participants had submitted for evaluation a test triple showing a descending series of numbers. A new binomial dependent variable was defined (presence or absence of the critical descending triple in the series of test triples), and a generalized linear model (logit link function, binomial family) was conducted based on these data. The main effect of Condition emerged (*χ*^2^(1, *N* = 120) = 23.376, *p* < .001; *R-squared* = 0.176; *power* (*alpha* = 0.05) = 0.998). As shown in Figure 4, the proportion of participants who submitted a descending test triple was significantly higher in the training condition (95%) compared to the control condition (60%).

We then studied when the relevant property appeared in the series of test triples in the two conditions. We addressed this aspect in two ways, in both cases focusing only on the subset of our initial sample consisting of those participants who, at some point, submitted a descending triple.

**Figure 5:**
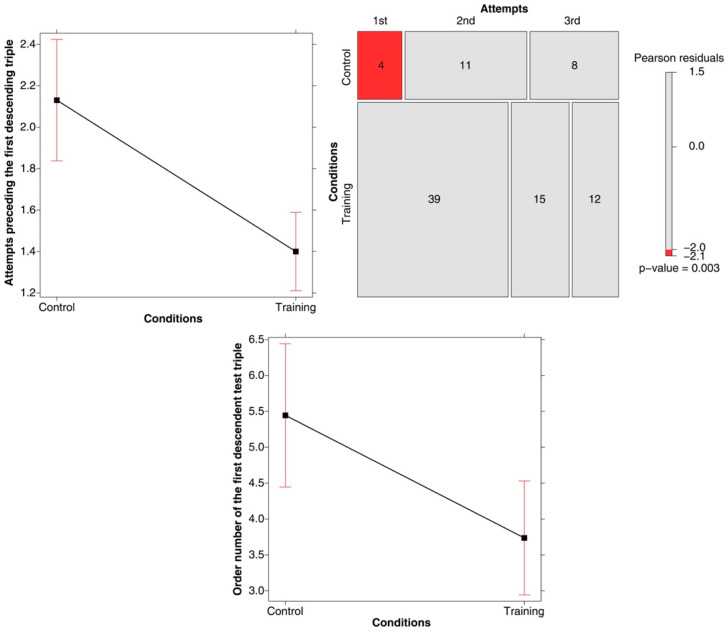
(**Top left panel**) Main effect of Attempt prior to which a descending triple was submitted for the first time by the participants in the two Conditions. Error bars represent the 95% confidence interval. (**Top right panel**) Mosaic plot of the number of test triples including a descending triple (produced prior to the first, second, and third attempts) in the two Conditions. (**Bottom graph**) Main effect of Order number of the first descending test triple submitted in the two conditions. Error bars represent the 95% confidence interval.

A first analysis (a linear model with Condition as a fixed effect) was conducted to ascertain whether a descending triple was submitted on the first, second, or third attempt at guessing the rule. The main effect of Condition was significant (*F*(1, 76) = 17.393, *p* < .001; *R-squared* = 0.186; *power* (*alpha* = 0.05) = 0.999). As shown in the top left panel of Figure 5, in the training condition, the participants submitted at least one descending triple for evaluation earlier than those in the control condition.

The mosaic plot presented in the top right panel of Figure 5 shows that in the training condition, a reference to at least one descending triple was found in 39 test triples (out of a total number of 60) submitted prior to the 1st attempt, in 15 of the test triples prior to the 2nd attempt, and in 12 of the test triples prior to the 3rd attempt. Conversely, in the control condition, a reference to at least one descending triple was found in only 4 of the test triples prior to the 1st attempt, in 11 of the test triples prior to the 2nd attempt, and in 8 of the test triples prior to the 3rd attempt. As evidenced by the colored tiles, the differences between the two conditions concern, in particular, the number of cases in which the critical property appears in the series of test triples produced before the first attempt at guessing the rule.

A second way to explore how long it took the participants to submit a descending test triple was to list all of the test triples produced during the experiment by each of the participants and then record and code the point at which a descending triple first appeared (we call this the Order number of the first descending triple submitted). This analysis was only conducted on the subset of participants who actually produced a descending triple. A linear model was then carried out on the order number of the first descending triple, with Condition as a fixed effect. It was found that a descending triple came up after a greater number of test triples in the control condition compared to the training condition (*F*(1, 91) = 7.072, *p* = 0.009; *R-squared* = 0.072; *power* (*alpha* = 0.05) = 0.857)—see Figure 5 (bottom panel).

**Figure 6:**
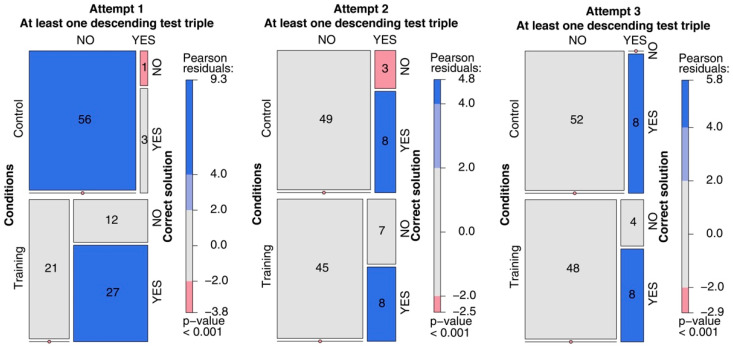
Mosaic plot of the association between producing (or not producing) at least one descending triple and guessing the correct rule in the subsequent attempt. This analysis refers to the first, second, and third attempts separately in both the training and control conditions.

**(4) Using the critical property to solve the task.** The three mosaic plots in Figure 6 enabled us to investigate whether there was an association between the participants testing a descending triple and their identifying the correct rule in the subsequent attempt. As the blue tiles confirm, there is an association between having tested at least one descending triple before attempting to guess the rule and then guessing correctly. This holds for all three attempts in the training condition and for the second and third attempts in the control condition. For the first attempt in the control condition, the symmetric relationship holds; that is, not submitting a descending triple was associated with not guessing the rule more frequently than might be expected by chance.

**Figure 7:**
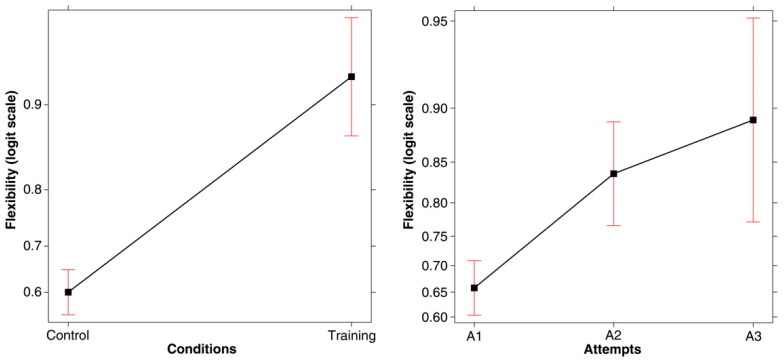
(**Left panel**) Main effect plot of Condition on Flexibility (i.e., the proportion of triples testing different aspects over triples testing the same aspect). (**Right panel**) Main effect plot of Attempts on Flexibility. In both graphs, error bars represent the 95% confidence interval.

**(5) Flexibility.** This refers to whether the participants avoided reiteration of similar types of test triples. In order to collect further information on how the training might have modified their testing procedure, we explored whether the test triples produced before each attempt tested the same property or, conversely, focused on different properties. The latter strategy would indicate that participants were exploring a number of different alternatives in the hypothesis testing phase (i.e., they were flexible). We expressed the degree of flexibility proportionally; that is, the number of test triples that focused on different aspects of the seed triple over the number of test triples that focused on the same aspect. This means that the higher the value, the greater the degree of Flexibility. The classification was made by two independent judges with a good inter-rater agreement (Cohen’s kappa coefficient was equal to 0.89). A generalized linear mixed-effect model was carried out on these proportional values (family Poisson, logit link function) with Condition and Attempt as fixed effects and Participants as a random effect. Two main effects emerged for Condition (*χ*^2^(1, *N* = 120) = 65.994, *p* < .001) and Attempt (*χ*^2^(2, *N* = 120) = 28.798, *p* < .001; Conditional *R-squared* = 0.297; *power* (*alpha* = 0.05) = 0.998). Flexibility was significantly higher in the training condition than in the control condition (see left panel of Figure 7, which displays the main effect plot of Condition, with data expressed in logit). The row index of flexibility was 0.901 ± 0.164 in the training condition and 0.642 ± 0.296 in the control condition. Furthermore, in both conditions, flexibility was higher for the second and third attempts than for the first attempt (see the right-hand panel of Figure 7; Bonferroni’s post hoc attempt 1 vs. 2: *z-ratio* = −3.686; *p* = .0007; attempt 1 vs. 3: *z-ratio* = −2.882; *p* = 0.011).

## 4. Final Discussion

In this final section, we first focus on the main empirical outcomes of the study and discuss related aspects, including its limitations and possible ways to address these in future research. We then consider some theoretical issues arising from these findings.

The results of this study showed significant improvement in performance under the training condition compared to the control condition in terms of the proportion of participants who succeeded in discovering the rule (78.33% vs. 36.66%, Hypothesis 1). There was also an improvement in efficacy relating to the search process after training (Hypothesis 2). Although it took, on average, longer to find the correct rule under the training condition, the participants guessed the rule more often on the first attempt than those in the control condition (57.4% versus 13.63%). Thus, while the search took longer, it was more effective in terms of the results since fewer incorrect guesses (attempts) were made.

An analysis of whether the participants included a series of descending numbers in the test triples submitted to the experimenter for evaluation (Hypothesis 3) helped us to understand the reason for the abovementioned improvements. In the control condition, there were fewer test triples involving ascending/descending series of numbers, and in any case, these occurred at a later stage (i.e., after a greater number of test triples). A joint analysis that investigated the association between submitting a descending test triple and finding the correct rule confirmed that descending test triples more frequently led to success.

An additional analysis of the test triples showed that training resulted in less reiteration of triples testing the same criterion and, conversely, fostered flexibility in testing various properties (Hypothesis 4).

This study aimed to provide a first assessment of the efficacy of training participants to think in opposites when performing Wason’s 2-4-6 discovery task. There is already evidence that this strategy facilitates visuospatial problem-solving (Bianchi et al. 2020; Branchini et al. 2015a, 2015b, 2016, 2021), but it has never been applied to Wason’s task. The results summarized above confirm that, in this task also, training positively impacted the participants’ ability to find the correct solution. Moreover, the differences that emerged from the analyses of the test triples gave some indications about the way in which training might have modified the hypothesis testing procedure. This is an interesting result that merits further investigation.

In particular, a variation of the experimental condition is needed to better understand the effects of the various phases involved in the training given, during which the participants (a) were invited to focus on the various different characteristics of the triples, (b) were explicitly prompted to ascertain whether or not each characteristic was crucial, and finally (c) were explicitly requested to identify the opposite of each characteristic as a specific way of testing it. Each of these phases might potentially have contributed to the differences found between the training and control conditions. In order to disambiguate the role of thinking in opposites (i.e., the third step of the training) from the other two preliminary phases, further studies are needed in which participants are exposed to training sessions that include the two initial phases but not the third one. In the meantime, it is, however, possible to speculate based on previous literature.

The results of the studies on visuospatial problem-solving cited previously (see in particular Bianchi et al. 2020) suggest that focusing in an analytical way on the characteristics of a problem (i.e., phase one) might not be enough to explain the improvements in performance that emerged in the present study. In the context of visuospatial problem-solving, a certain amount of awareness regarding the use of opposition as a strategy turned out to be critical. A simple, analytical list of all of the characteristics of the problem at hand did not have the same results as an explicit strategy involving the identification of the opposites of these characteristics.

We might expect that an explicit prompt to focus on each characteristic one at a time (i.e., the second phase of the present training condition) would have a positive impact from two points of view. First, it might help reasoners to avoid reiteration, that is, repeatedly testing the same hypothesis. If we consider that the process of formulating and testing a hypothesis depends on the evidence and data at the participant’s disposal (Girotto and Gonzalez 2000; Ragni et al. 2018), then identifying each individual characteristic might make the reasoners realize that there are as many alternative hypotheses as there are properties. Secondly, identifying the individual characteristics one at a time might prevent the reasoners from neglecting some aspects, for example, the fact that the three numbers are ascending, independently of other characteristics regarding the intervals between the numbers. The more evident a property is, the easier it should be to notice and select it as a candidate for testing. This might correct the default trend to take for granted that the rule concerns an ascending series of numbers without challenging it and focus on other characteristics (such as whether the series consists of odd or even numbers and whether the intervals between the numbers are equal or not). It would be interesting to understand whether the range of different alternatives explored by participants trained to focus on each individual characteristic of the series of numbers varies compared to that of those who are exposed to a training program that also includes the third phase (i.e., thinking in opposites).

We cannot exclude the possibility that there is a positive effect on performance resulting from the application of these two phases, but we feel that a strategy that involves thinking in opposites (i.e., the third phase) adds a plus. We will try to explain this last statement by referring to two theoretical ideas that were briefly mentioned in the introduction, which are the idea of contrast class and the idea of the iterative counterfactual model. Our training program was not inspired by these ideas, and in this sense they do not constitute the theoretical foundation for this study. As we have already made clear, the present research was motivated by a desire to test the effects of a training program that had previously been used successfully with visuospatial problem-solving on a different task (Wason’s discovery task). However, the consistent findings emerged from prompting participants to think in opposites in these two different contexts (i.e., visuospatial problem-solving and Wason’s discovery task) lead one to wonder whether and how this technique might relate to both the idea of contrast class and the idea of the iterative counterfactual model.

The idea that thinking in opposites can support hypothesis testing has already, in a sense, been mentioned in Gale and Ball’s paper (Gale and Ball 2012). The authors did not specifically aim to test the efficacy of a prompt to think in opposites. Their aim was to test the contrast class account of the dual goal task facilitation effect. In particular, their hypothesis was that reasoners are acutely sensitive to the dimensions on which oppositional hypotheses, such as DAX and MED, lie. Because of this, the triple 6-4-2 provided for the MED rule was expected to be more functional than the other triples (i.e., 4-4-4 and 9-8-1) in that it alerted the participant to the possibility that the two rules, MED and DAX, might concern the descending-ascending dimension. Their findings confirmed their prediction. No other implications of these findings were derived by the authors beyond the specific condition considered (i.e., the dual goal task). However, in the final discussion, they connected the idea of contrast class to the iterative counterfactual model by Oaksford and Chater (1994a, 1994b; Oaksford 2002) as a useful theoretical context to account for their results. This stimulated us to wonder whether and how the training used in our study might be connected to the iterative counterfactual model.

According to this model (Oaksford and Chater 1994a, pp. 158–63), a reasoner engaged in the standard version of Wason’s task would start from an initial working hypothesis based on the seed triple that takes into account all of the salient information available (Cherubini et al. 2005). This first hypothesis might, therefore, be “even numbers in ascending order at intervals of two”. Based on the strategy suggested by Tschirgi (1980), the reasoner would then formulate and test a second, counterfactual hypothesis that differs from the first hypothesis with respect to only one relevant dimension. This second hypothesis might be, for instance, “odd numbers in ascending order at intervals of two”. Based on the feedback received, the reasoner would repeat this strategy and, each time, formulate a new hypothesis in addition to a related counterfactual hypothesis. This would help them to explore the boundaries of the rule progressively.

In presenting their model, Oaksford and Chater (1994a) explain that it is meant to extend Farris and Revlin’s (1989a, 1989b) counterfactual strategy beyond the context of falsification to the context of hypothesis generation. The training to think in opposites in the present study potentially acts on both levels relating to the way in which hypotheses are generated—i.e., the context of discovery, according to Oaksford and Chater (1994a, p. 150)—and the way in which they are tested—i.e., the context of falsification, according to Oaksford and Chater (1994a, p. 150). The first phase of the training (in which the participants are asked to identify all the characteristics of the seed triple) supports hypothesis generation. They focus on one feature at a time and wonder whether that is a salient characteristic. In other words, they use it to generate their hypothesis. For example, they might notice that the seed triple is made up of even numbers and generate the hypothesis that this characteristic is relevant. In the third phase of the training they think in opposites in order to test whether a series formed of odd numbers still fits in with the rule.

An idea that seems to be hinted at by Oaksford and Chater (1994a) in two passages of their paper is transformed by the technique of thinking in opposites into a precise, systematic procedure. One passage is a footnote wherein they comment on the following statement: “Farris and Revlin (1989a, 1989b) argue that subjects may be adopting a counterfactual strategy in the 2-4-6 task. On this strategy, a hypothesis is first generated based on some property of the seed triple, e.g., even numbers. The *complement* of this rule is then generated, i.e., odd numbers. This rule is assumed to be true and subjected to a confirmatory test” (Oaksford and Chater 1994a, p. 154). In the footnote, they specify: “Note that ‘complement’ is being used imprecisely in this context. The set union of the set of even natural numbers and the set of odd natural numbers yields the set of all natural numbers (…). However, the set union of the set of even numbered triplets and the set of odd numbered triplets does not yield to the set of all number triplets (…). Hence, ‘complement’ is not meant as logical or set theoretic complement (…). In this context ‘number theoretic *opposite*’ may have been a more precise term” (Oaksford and Chater 1994a, p. 154, footnote 7; all italics in these citations are original). They go back to this distinction a few lines later when they state that “while it is not the case that all complements are opposites, all opposites are complements. (…) the value of an opposite in this context is that they are derived from familiar *antonymic* structures in language that suggest obvious triplets to try out as instances of what is not in H. Contrast, for example, generating an instance of the complement of the Hypothesis H1: *even numbers*, and of the hypothesis H2: that *the number triples are derived from the Fibonacci series*. Only the former has an opposite defined by its complement in the domain of natural numbers (…) This makes for ready identification of an appropriate instance of the complement of the hypothesis under test. So if you want an instance of a number that is *not*-even, then an odd number is the best bet.” (Oaksford and Chater 1994a, p. 156).

The training used in the present study might be seen as a development of these two specific observations. It immediately replaces the idea of “alternative to x” and “not-x” (negation) with the idea of “opposite to x” for a given property. There are benefits and costs resulting from this. The cost is that, logically, the complement set is more extensive than the subset that includes opposites. The benefits are cognitive. The first relates to the fact that the idea of opposites is more intuitive than other more technical or logical definitions, such as counterfactuals or complement sets, and the bond between imagining alternatives in everyday counterfactual thinking and opposites is primal (see Section 1.2). This means that thinking in opposites can easily become a deliberate strategy within everyone’s reach to produce alternatives for initial stimuli—visual representations, concepts, numerical series, etc. A second benefit is that negation, cognitively, entails the mental construction of a situational model that implies a variation from the (negated) situation along one or more dimensions with opposite poles. This has been effectively argued (Kaup et al. 2007; Branchini et al. 2021, p. 3; Schroyens et al. 2001, pp. 152–58) and empirically demonstrated (see, for instance, Bianchi et al. 2011a; Kaup et al. 2006; Paradis and Willners 2006). For example, “the water is not hot” makes us think of water that might be warm, lukewarm, or cool, just as “the door is not open” brings to mind a door that has a degree of aperture along the open-close dimension. In other words, the bond between opposites and negation is cognitively primal. For all of these reasons, a strategy hinting at thinking in opposites might facilitate people to explore alternatives and counterfactuals in an intuitive but systematic way (even though this would not cover all the logical alternatives). These considerations might also be relevant to research concerning conditional inferences implying negation—for instance, studies that investigate whether still unexplained effects of implicit versus explicit negation in conditional inferences might be explained by structural aspects of the contrast set activated by negation in reasoners’ minds (e.g., Schroyens et al. 2000; Vance and Oaksford 2021).

Because opposites, by definition, consist of pairs of properties, they offer a method of opening up the space within which the search is carried out while at the same time giving precise directions. This combination of openness and boundaries fits in with the requisites of an effective cognitive heuristic (Öllinger et al. 2013; see also Branchini et al. 2021, p. 9).

A training program, such as the one described in this paper, also has the advantage that it is not specific to the content of the problem. Indeed, the training referred to shapes, and the participants were asked to transfer the strategy to numbers. Similarly, in the previously mentioned studies on visuospatial problem-solving (Bianchi et al. 2020; Branchini et al. 2015a, 2015b, 2016, 2021), the training referred to three visual problems, and the strategy was then applied to other visual problems. The possibility that analogical transfer might be part of the process does not weaken the premises of training, which presupposes that the reasoners will find it easy to think in opposites in a variety of situations. The limits to the fruitful application of this strategy are yet to be empirically explored (see Branchini et al. 2021; Bianchi and Branchini 2023).

The utility of thinking in opposites in individual versus group settings is another aspect that needs to be explored further. In the present study, the participants worked individually. Conversely, Branchini et al. (2016) found that a prompt to think in opposites only improved performance when the strategy was applied in a small group but not in an individual condition. Similarly, Augustinova (2008) found that a falsification attitude worked better with small groups. The tasks used in these studies are all different, and therefore, the explanation of these contradictory results might concern the kind of search process activated by each specific task. In light of these considerations, a limitation of the present study is that a group condition was not included in addition to the individual condition. This may have allowed us to verify whether a suggestion to think in opposites would impact performance in Wason’s discovery task in similar ways depending on if the strategy is applied in individual or group settings and to look deeper into the dynamics underlying hypothesis generation and testing under these two conditions.

One final consideration relates to the fact that the percentage of rules guessed correctly on the first attempt was very similar in our study (57.4%) and Rossi’s study (Rossi et al. 2001) (54%) in which a counterfactual suggestion was used. Assuming this means that the two strategies have a similar facilitating effect, it would, in any case, be interesting to explore whether thinking in opposites is perceived by reasoners to be simpler from a phenomenological point of view than from thinking counterfactually since the latter involves a dual process at a metacognitive level (Ackerman and Thompson 2018; Thompson 2009; Thompson et al. 2011, 2013; Thompson and Johnson 2014). A good deal of further research is required.

## Data Availability

The raw data set was deposited, under creative commons attribution license, in the IEEE data repository, which is available online at the following link: https://dx.doi.org/10.21227/gpe8-2q64.
